# Overexpression of human Atp13a2^Isoform-1^ protein protects cells against manganese and starvation-induced toxicity

**DOI:** 10.1371/journal.pone.0220849

**Published:** 2019-08-08

**Authors:** Janet Ugolino, Kristina M. Dziki, Annette Kim, Josephine J. Wu, Bruce E. Vogel, Mervyn J. Monteiro

**Affiliations:** 1 Biochemistry and Molecular Biology Graduate Program, University of Maryland School of Medicine, Baltimore, Maryland, United States of America; 2 Center for Biomedical Engineering and Technology, University of Maryland School of Medicine, Baltimore, Maryland, United States of America; 3 Department of Anatomy and Neurobiology, University of Maryland School of Medicine, Baltimore, Maryland, United States of America; Northeastern Ohio Medical University, UNITED STATES

## Abstract

Mutations in *ATP13A2* cause Kufor-Rakeb syndrome (KRS), a juvenile form of Parkinson’s disease (PD) with dementia. However, the mechanisms by which mutations in *ATP13A2* cause KRS is not understood. The mutations lead to misfolding of the translated Atp13a2 protein and its premature degradation in the endoplasmic reticulum, never reaching the lysosome where the protein is thought to function. Atp13a2 is a P-type ATPase, a class of proteins that function in ion transport. Indeed, studies of human, mouse, and yeast Atp13a2 proteins suggest a possible involvement in regulation of heavy metal toxicity. Here we report on the cytoprotective function of Atp13a2 on HeLa cells and dopamine neurons of *Caenorhabditis elegans (C*. *elegans)*. HeLa cells stably overexpressing V5- tagged Atp13a2^Isoform-1^ protein were more resistant to elevated manganese exposure and to starvation-induced cell death compared to cells not overexpressing the protein. Because PD is characterized by loss of dopamine neurons, we generated transgenic *C*. *elegans* expressing GFP-tagged human Atp13a2 protein in dopamine neurons. The transgenic animals exhibited higher resistance to dopamine neuron degeneration after acute exposure to manganese compared to nematodes that expressed GFP alone. The results suggest Atp13a2 ^Isoform-1^ protein confers cytoprotection against toxic insults, including those that cause PD syndromes.

## Introduction

Parkinson’s disease (PD) is a progressive neurodegenerative disorder characterized by bradykinesia and tremor at rest [1]. The disease is associated with loss of dopamine neurons in the substantia nigra pars compacta. Besides age, which is a major risk factor for PD, mutations in several genes are linked to the cause of the disease [2, 3]. Additionally, a small number of cases have been linked to exposure to certain environmental toxins like pesticides and heavy metals [4, 5]. A particularly interesting connection linking genetic and environmental etiology of PD was the discovery that mutations in *ATP13A2* cause early-onset PD [6].

Mutations in *ATP13A2* cause juvenile parkinsonism with dementia, also known as Kufor-Rakeb syndrome (KRS) [6]. The *ATP13A2* gene encodes a protein that shares strongest homology with the P-type ATPase superfamily of ion pumps [7, 8]. Indeed, studies of Atp13a2 proteins in humans, mouse and yeast, all suggest Atp13a2 is somehow involved in regulating metal ion homeostasis. For example, *ATP13A2* knockout mice (KO) administered with manganese chloride had increased lipofuscinosis accumulation as well as manganese and iron accumulation in the brain compared to similarly treated wild type mice [9, 10]. Furthermore, knockdown of the Atp13a2 protein in human cells, or its yeast ortholog, sensitized the cells to heavy metal toxicity, particularly manganese and zinc, supporting the idea that Atp13a2 regulates transport of heavy metals [11–14]. Similar findings were found using patient-derived cells carrying *ATP13A2* mutations [14, 15]. In accord with its protective function, overexpression of human Atp13a2 protects cells against zinc and manganese-induced toxicity, although protection against manganese toxicity was not universally observed [14, 16]. A role in manganese protection, if correct, could be important in the pathogenesis of PD because high exposure to manganese has been implicated in the development of manganism, a PD-like syndrome [5].

There is evidence to suggest Atp13a2 protein may play a wider role in cytoprotection other than detoxification of heavy metals. For example, overexpression of human Atp13a2 has been shown to suppress toxicity of α-synuclein aggregates in primary rat and human neurons, while knockdown or knockout of the protein in cells or mice increases α-synuclein aggregation and induces proteotoxic stress [9, 12, 17–19].

The Atp13a2 protein localizes to lysosomes [6, 20–22]. By contrast, ATP13a2 proteins containing Kufor-Rakeb Syndrome disease-causing mutations fail to reach the lysosome and are instead prematurely degraded in the endoplasmic reticulum (ER) by the ER-associated degradation pathway [6, 21, 22]. Interestingly, fibroblast cells derived from patients carrying *ATP13A2* mutations have reduced staining of Atp13a2 protein in lysosomes [23] suggesting that the mutations cause PD syndromes from loss of Atp13a2 function in the lysosome. Apart from loss-of-function mutations that cause Kufor-Rakeb Syndrome, several other mutations in *ATP13A2* have been identified involved in a variety of other devastating diseases, including neuronal ceroid lipofuscinosis, PD and amyotrophic lateral sclerosis [24, 25]. The emerging evidence from these and other studies all point to an important role that Atp13a2 proteins play in lysosomal function and human health.

The lysosome is the site where cellular debris is degraded by hydrolytic enzymes and plays an instrumental role in autophagy by fusing with autophagosomes. This process is particularly important during times of nutrient deprivation because the lysosome can recycle cellular components for energy. Interestingly, defects in lysosome function and autophagy have been implicated in neurodegenerative diseases, including PD [26, 27].

Here, we investigated whether wild type human Atp13a2^Isoform-1^ protein plays a role in cytoprotection in HeLa cells and dopamine neurons of *C*. *elegans*. We show that overexpression of the protein protects both types of cells against manganese toxicity. We also show that consistent with its localization in lysosomes, Atp13a2 overexpression protects HeLa cells against starvation-induced cell death, when lysosome function becomes critical. Our results suggest Atp13a2^Isoform-1^ protein plays an important role in cytoprotection against different cellular insults.

## Materials and methods

### Generation of Atp13a2 stable cell line and tissue culture studies

HeLa cells (ATCC CCL-2) were grown in DMEM supplemented with 10% FBS at 37°C with 5% CO_2_. To generate the stable expressing Atp13a2 cell lines, HeLa cells were cotransfected with 1 μg neomycin expression vector (pSV2neo) and 10 μg V5-tagged Atp13a2^Isoform-1^ expression plasmid DNA [22] using the calcium phosphate co-precipitation method. Twenty-four hours after transfection, cells were trypsinized, diluted 10-fold with DMEM and replated. The next day, selection media (DMEM + 700 μg/ml G418) was added to the cells. Cells were maintained in selection media, which was replaced every three to four days. Ten days later, individual colonies were trypsinized and replated into wells of a 24-well plate containing fresh selection media and allowed to grow for another 5 days after which the colonies were expanded to a 6-well dish in duplicate. After 48 hours, lysates were collected and expression of Atp13a2 was determined by immunoblotting using an antibody to V5. Colonies that stably expressed Atp13a2 were maintained in selection media for future experiments.

### Cell death assays

HeLa cells or the V5-tagged Atp13a2-expressing HeLa cells were plated at equal density and allowed to incubate for 24 hours for cell death analysis. For starvation experiments, the cells were incubated in starvation media (1 x PBS + 0.9 mM CaCl_2_ and 0.9 mM MgCl_2_) [28] for 16 hours prior to analysis. For manganese toxicity experiments, cells were treated with 500 μM or 800 μM MnCl_2_ (Sigma-Aldrich) by addition of MnCl_2_ to the DMEM medium for 16 or 24 hours prior to cell death quantification. Cell death was quantified by counting the number of cells that had highly condensed or fragmented DNA by visual examination of images captured under the microscope following incubation of the cells with the nuclear dye Hoechst 33342 (Sigma-Aldrich) for 15 minutes at a final concentration of 0.5 μg/ml prior to viewing. The cells were viewed live with a Leica DM IRB microscope using a Leica PL FLUOTAR 10x/0.30 Ph 1 lens and images captured with a Hamamatsu digital camera C8484 using iVision-Mac software (BioVision Technologies). At least 500 cells were counted for cell death analysis per trial.

For florescence staining of cells, HeLa cells were grown on glass coverslips overnight and then fixed with 4% paraformaldehyde for 30 minutes prior to being stained with primary and secondary antibodies as described previously [22]. Slides were viewed with a Leica DM IRB microscope using a Leica PL FLUOTAR 40X/0.70 Ph2 lens and images captured with a Hamamatsu digital camera C8484 using iVision-Mac software.

### SDS-PAGE and immunoblotting

Protein lysates were made by resuspending HeLa cells or nematodes that had been pelleted by centrifugation with about 50X their volume of protein lysis buffer (0.5% SDS, 0.5% NP40, 0.5% sodium N-lauroylsarcosine, 50 mM Tris pH 6.8, 150 mM NaCl, 20mM EDTA, 1mM EGTA, 25 mM sodium fluoride, 1mM sodium orthovanadate, 1 mM Pefabloc (AEBSF, Roche, Indianapolis, IN, USA), 1 mM leupeptin and 1 mM aprotonin) [29]. The lysates were sonicated for 3 min with a Branson Sonifier 450 attached with a 1/8-inch microtip (Branson Ultrasonics, Danbury, CT). Protein concentrations were then measured using the bicinchoninic acid assay (Thermo Fisher Scientific, Waltham, MA, USA) and appropriate amounts of the lysate were mixed with SDS sample loading buffer and heated for 5 min at 100°C prior to gel loading. Equal amounts of protein were separated by SDS-PAGE on either 8.5 or 10% polyacrylamide gels and the separated proteins were then transferred onto 0.45 μm PVDF membranes (Millipore, Billerica, MA, USA) for 3 h at 200 mA using the BioRad Mini Transfer Blot System (BioRad, Berkeley, CA, USA). The membranes were blocked for 1 hr in 5% bovine serum albumin in 1 x TBS buffer (10 mM Tris pH 7.5, 150 mM NaCl) and then incubated at 4°C overnight with appropriate primary antibodies in fresh blocking solution. The following primary antibodies were used: rat monoclonal anti-Lamp2 (#ABL-93, Santa Cruz Biotech, Santa Cruz, CA, USA), rabbit polyclonal anti-V5 (#AB3792, Millipore-Sigma, St. Louis, MO, USA), goat polyclonal anti-actin (#sc-1615, Santa Cruz Biotech), rabbit polyclonal anti-LC3 A/B (#4108, Cell Signaling, Danvers, MA, USA), rabbit polyclonal ant-p97/VCP, anti-GFP and anti-mRFP (all produced in house and verified by reaction with recombinant proteins). Antibody binding was detected by chemiluminescence following incubation with appropriate secondary horseradish peroxidase conjugated antibodies (all from Thermo Fisher Scientific, Waltham, MA) using the SuperSignal West Pico system (Thermo Fisher Scientific). The luminescence signals were captured using a Fluoro-Chem M imager (Protein Simple, Santa Clara, CA, USA) and the intensity of different bands was quantified using AlphaView software (Protein Simple).

### Autophagic flux assay

Cells were grown in DMEM with 4.5 g/l glucose (#10-107-CV, Corning, Corning, NY, USA) supplemented with 10% FBS (Sigma, St. Louis, MO) at 37°C with 5% CO_2_. For autophagic flux assay, cells were plated at equal densities and the next day the cultures were washed 3 times with DMEM without glucose (#11966–025, Gibco Life Sciences, Thermo Fisher Scientific) and treated with, or without bafilomycin A1 (Enzo Life Sciences, Farmingdale, NY) at a concentration of 200 nM, for either 0, 2 or 4 h. Cells were then washed in ice-cold 1 x standard PBS twice and protein lysates were prepared as described above. Equal amounts of proteins were immunoblotted for the proteins as shown in the figures. The results shown were repeated three times, producing similar results.

### Measurement of autophagosome maturation

The maturation of autophagosomes into autolysosomes was measured using the tandem-tagged pDest-mCherry-LC3 reporter construct [30]. For this assay, cells from the Atp13a2-V5 stable line or the parental HeLa cell line were plated on coverslips and grown in DMEM with 4.5 g/l glucose at 37°C with 5% CO_2_. The next day the cells were transfected with the GFP-mCherry-LC3 reporter plasmid using the calcium phosphate coprecipitation method [31]. The following day the cells were washed 3 times with DMEM without glucose and incubated for either 0, 2 or 4 h, with, or without, bafilomycin A1, essentially as described above. Coverslips were removed at appropriate time intervals and fixed in 4% paraformaldehyde in 1 X PBS for 15 min and then stained with 4’6-diamino-2-phenylindole (DAPI) in 1 x PBS for 5 min. The coverslips were then washed 3 times in 1 x PBS and mounted on glass slides. GFP, mCherry, and DAPI fluorescent images of individual cells were captured with a 100 x/1.25 Acroplan oil-immersion lens using a Zeiss Axiovert 200 microscope attached with a Hamamatsu Digital Camera C4742-95 (Hamamatsu Corp, Bridgewater, NJ, USA), using Simple PCI imaging software (Simple PCI, Cranberry Township, PA, USA). The digital images were merged using iVision software (Biovision Technologies, Exton, PA, USA). The percent of acidified autophagosomes in cells at each time point was determined by calculating the proportion of puncta exhibiting mainly mCherry fluorescence and little GFP fluorescence (by examining the merged fluorescence images) relative to the total number of fluorescent puncta in the same cell. The data shown is derived from quantification of at least 5 different cells.

### *C*. *elegans* methods and generation of the line expressing Atp13a2-GFP in dopamine neurons

*C*. *elegans* were grown and maintained using standard techniques (Brenner, 1974). Three lines were used in the study. One of them expressing GFP in dopamine neurons (BZ555: egIs1 [dat-1p::GFP] was obtained from the Caenorhabditis Genetics Center (University of Minnesota), whereas we generated the two other lines, one of which coexpressed GFP and mRFP, and the other that coexpressed GFP-tagged human Atp13a2^Isoform-1^ and mRFP, in dopamine neurons. To express Atp13a2-GFP in dopamine neurons of *C*. *elegans* the entire open reading frame (ORF) of Atp13a2^Isoform1^, fused in frame with GFP at the C-terminus, was subcloned from our CMV expression plasmid [22] downstream of the dat-1 promoter (pdat: kindly provided by Dr. Randy Blakely, Vanderbilt University). A 1:1 mixture of the two plasmids was injected into the gonads of early-adult N2 hermaphrodites and transgenic F1 progeny were picked onto fresh plates. F2 progeny that expressed both mRFP and Atp13a2-GFP in dopamine neurons were used to establish the line that was used for the manganese toxicity assays. Likewise, pdat-1 driven mRFP and GFP expression constructs were coinjected and nematodes coexpressing both fluorescent proteins were isolated and used for the manganese toxicity assays.

### Manganese toxicity assays of *C*. *elegans*

Toxicity assays were conducted using synchronized L1 stage nematodes. For synchronization, gravid adults were harvested from three to five 60 mm plates with M9 buffer (3 g KH_2_PO_4_, 6 g Na_2_HPO_4_, 5 g NaCl, 1 ml of 1 M MgSO_4_ per liter of water). The pooled nematodes were centrifuged at 3,000 rpm for 3 min and the supernatant was discarded. The nematodes were resuspended in 5 mls of alkaline hypochlorite solution (made by mixing 20 mls of Clorox house bleach (6% NaOCl) with 2 g of NaOH and 30 mls of water) and incubated for 5 min in the solution with gentle agitation. The released eggs were pelleted by centrifugation and washed three times with M9 buffer. They were then resuspended in 5 ml of M9 buffer and gently shaken overnight at room temperature (RT). The following morning, the hatched L1 larvae were spun at 3500 rpm for 5 min, and the supernatant was removed. The larvae were re-suspended in 6 mL of 85 mM NaCl solution and divided into six microcentrifuge tubes. The tubes were centrifuged at 7,000 rpm for 3 min and supernatant discarded. The nematodes were gently resuspended in 1 ml of 85 mM NaCl containing varying concentrations of MnCl_2_ (0, 50, 150, 250, 350, or 500 mM) and incubated for 1 h at RT with periodic mixing. The tubes were then spun at 7,000 rpm for 3 min minutes and washed 3 times with 1 mL of 85 mM NaCl. After the last wash, the larvae were plated onto the NGM agar plates seeded with OP50 bacteria. The nematodes were left for ~24 hours at RT and then examined by fluorescence microscopy to evaluate dopamine neuron degeneration.

### Classification of healthy and unhealthy nematodes

Dopamine neuron degeneration was examined by collecting nematodes growing on 60 mm plates with 200 μL of M9 buffer. Approximately 100 μL of this suspension and 50 μL of 3 mM Tetramisole hydrochloride (to immobilize nematodes) made in M9 buffer were then mixed and placed on a glass slide and covered with a glass coverslip. The slides were then examined immediately by fluorescence microscopy using a LEICA MZ16FA stereo microscope equipped with a LEICA PLANAPO 1.0x lens in combination with either a GFP 2 LEICA 10447295 filter for GFP fluorescence or a DSR LEICA 10447292 filter for RFP fluorescence and images captured with a Hamamatsu digital camera C8484 using iVision-Mac software. Colocalization was performed using iVision-Mac software. Nematodes were classified as being “healthy” if all four cephalic (CEP) dopamine neuron dendritic processes were visible and had bright and solid fluorescence, with the exception that some speckling in both the BZ555 and Atp13a2 lines was inherently present. Nematodes were classified as “unhealthy” if they had at least one dendritic process that was either patchy, or was speckled in appearance. They were “dotted”-looking, with bright speckles alternating with sections that were completely dark, suggestive of degeneration. For the BZ555 line we used GFP fluorescence to monitor degeneration whereas mRFP fluorescence was used for the Atp13a2 line and GFP::RFP lines.

### Statistical analysis

Student t tests using Microsoft Excel software was used for all statistical analysis for the cell death assays. Error bars represent ± the standard deviation of the mean (SDM). Two-way ANOVA analysis was conducted for the autophagy flux and nematode studies using GraphPad software (La Jolla, CA). P≤ 0.05 was considered to be statistically significant.

## Results

### Generation of HeLa cells stably expressing Atp13a2^Isoform-1^ protein

**Fig 1:**
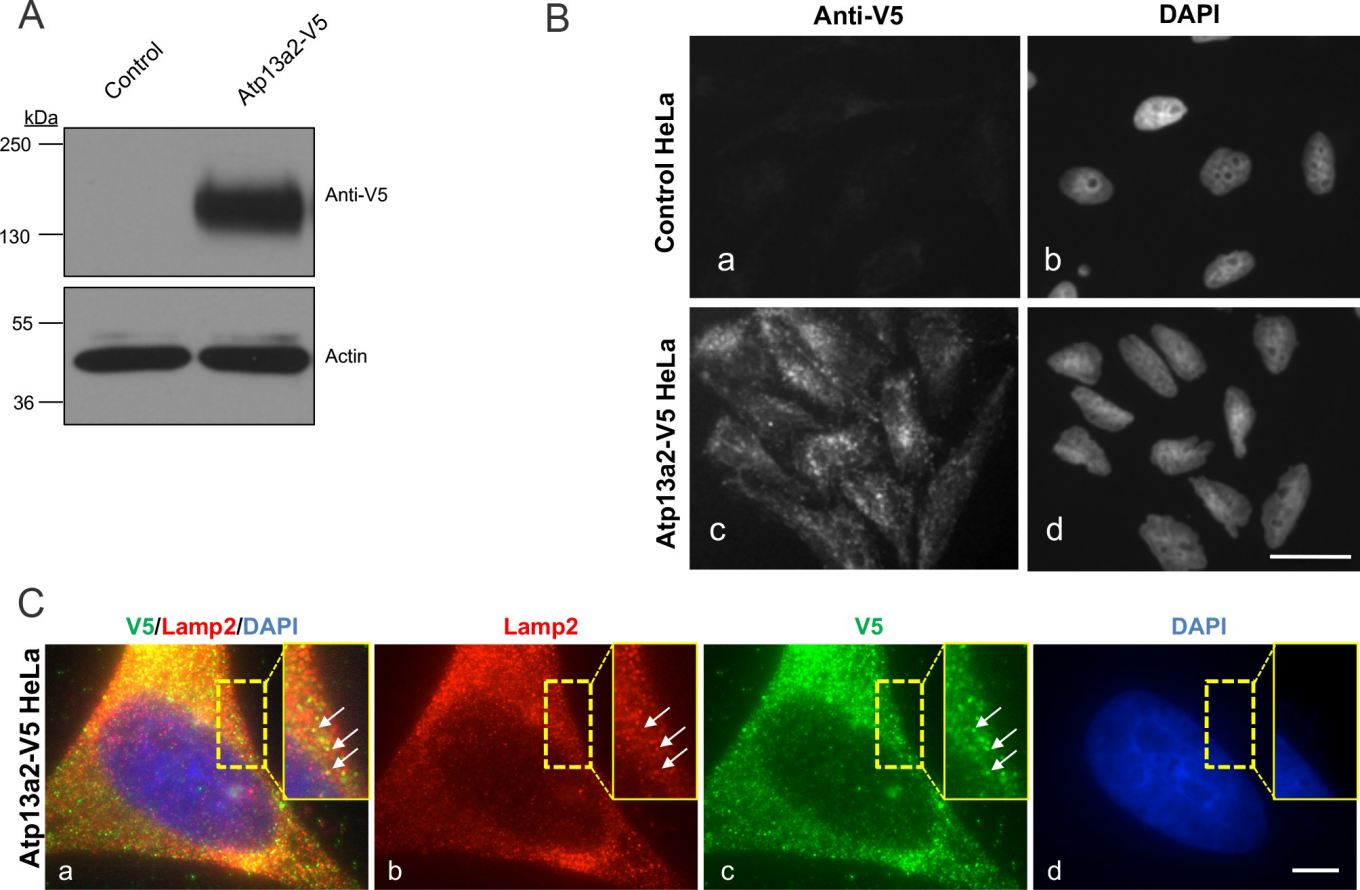
Isolation of a cell line overexpressing human Atp13a2. (A) HeLa cells stably expressing Atp13a2-V5 or the parental control HeLa cells were lysed and equal amounts of protein were probed with a V5 antibody to detect expression of the V5-tagged Atp13a2 protein by immunoblotting. Actin was used to verify protein loading. (B) Control HeLa cells (a and b) or HeLa cells stably expressing Atp13a2-V5 (c and d) were grown on coverslips and stained with the anti-V5 antibody followed by a FITC-conjugated secondary antibody and examined by immunofluorescence microscopy. The anti-V5 antibody decorated puncta in the stable cell line, which is consistent with localization of the Atp13a2-V5^Isoform-1^ protein to lysosomes. (C) HeLa cells stably expressing Atp13a2-V5 were double stained with anti-V5 and anti-Lamp 2 antibodies and their binding was detected with Alexa 488-conjugated and Alexa 594-conjugated secondary antibodies. The images shown are of the same cell imaged for the two different fluorescent proteins and DAPI, which was used to stain the nucleus. The result of merging of the three individual channels is also shown. The stippled box is a magnified portion of the cell to better illustrate the colocalization of V5 and Lamp 2 staining (white arrows). Bar, 10 μm.

To investigate the possible protective function of Atp13a2 protein, we generated HeLa cells stably overexpressing V5-tagged human wild type Atp13a2^Isoform-1^ protein (Fig 1). Previously, we showed that upon transient transfection of HeLa cells, the Atp13a2^Isoform-1^ protein localizes to lysosomes [22]. To confirm expression and localization of the V5-tagged Atp13a2 protein in the stable cell line we utilized immunoblotting and immunofluorescence microscopy. An immunoblot probed with anti-V5 antibody revealed a robust band of appropriate size for the V5-tagged Atp13a2 protein in the stable cell line, but not in the parental cells from which the cell line was derived (Fig 1A). Furthermore, immunofluorescence microscopy revealed that the V5-tagged Atp13a2 protein was localized to vesicular structures, consistent with the localization of the wild type Atp13a2 protein to lysosomes (Fig 1B) [22]. Indeed, double immunofluorescence microscopy for V5 and Lamp 2 protein staining indicated colocalization in numerous puncta, supporting Atp13a2-V5 tagged protein is localized to lysosomes (Fig 1C). However, there was considerable variation in the strength of the two signals, where some puncta were brighter for one protein than the other, the reason for which is unclear. Nevertheless, the results demonstrate successful generation of HeLa cells overexpressing Atp13a2 protein with localization to lysosomes.

### Expression of Atp13a2 protects cells from starvation-induced cell death

**Fig 2:**
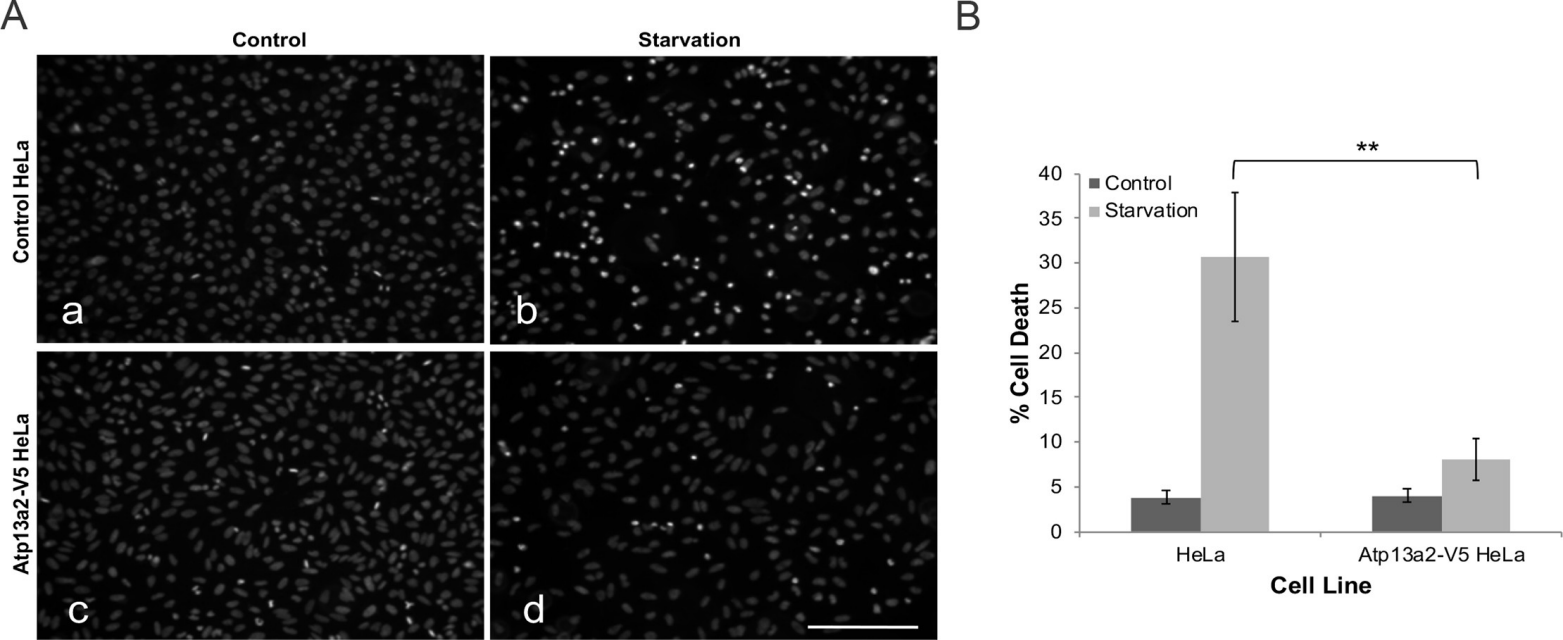
Overexpression of Atp13a2 protects cells against starvation-induced cell death. (A-B) HeLa cells or HeLa cells stably expressing V5-tagged Atp13a2 were cultured under normal growth conditions (a and c) or under starvation conditions (b and d) for 16 hours and then stained with the nuclear dye Hoechst 33342. Cell death was quantified by counting cells that had highly condensed/fragmented DNA based on the strong staining with the nuclear dye Hoechst 33342 (A) Representative Hoechst staining of control and starved HeLa cell nuclei. Bar, 100 μm. (B) Quantification of cell death in normal HeLa cells and the Atp13a2-expressing cell line grown under normal and starvation conditions. Graph is an average of three independent experiments and the data shown is the mean ± standard deviation of the mean (SDM). Cells expressing Atp13a2-V5 exhibited significantly lower cell death compared to normal HeLa cells after starvation (**p < 0.005).

Because lysosomes play a fundamental role in the survival of cells during nutrient deprivation, we examined whether the increased expression of Atp13a2 protein in lysosomes would protect HeLa cells from starvation-induced cell death. To address this question, we measured cell death in HeLa cells overexpressing Atp13a2 protein and the parental cells not overexpressing the protein when grown under normal growth conditions or incubated under starvation conditions for 16 hours (Fig 2A and 2B). The quantification revealed that the 16-hour starvation period increased cell death in the parental HeLa cells compared to cells cultured under normal conditions, as expected. By contrast, the cells stably overexpressing Atp13a2 were dramatically resistant to nutrient deprivation as cell death only marginally increased upon starvation. These results suggest that expression of Atp13a2 protects cells from starvation-induced cell death.

### Expression of Atp13a2 protects HeLa cells from manganese cytotoxicity

**Fig 3:**
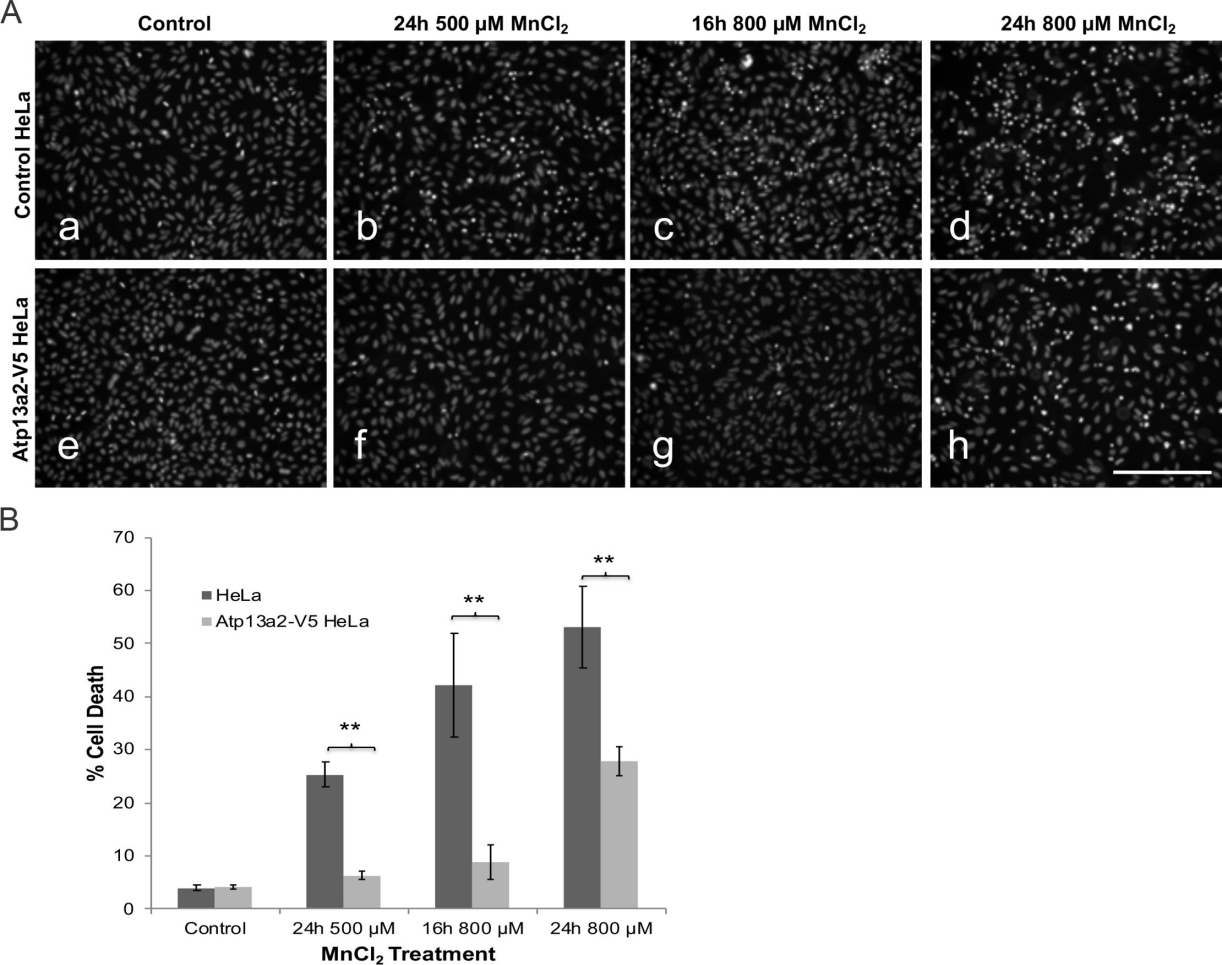
Overexpression of Atp13a2 protects cells from manganese cytotoxicity. (A-B) HeLa cells or HeLa cells stably expressing V5-tagged Atp13a2 were grown under normal growth conditions (a and e) or were exposed to 500 μM (b and f) or 800 μM MnCl_2_ for 16 or 24 hours (c, d, g and h) prior to cell death analysis. Cell death was quantified by Hoechst 33342 staining. (A) Representative Hoechst staining of HeLa cells after MnCl_2_ treatment. Bar, 100 μm. (B) Quantification of cell death. Graph is representative of three independent experiments and data is shown as mean ±SDM. Cells expressing Atp13a2 show a significant decrease in cell death compared to normal HeLa cells after exposure to MnCl_2_. (**p < 0.005).

Toxicity studies have suggested that Atp13a2 proteins protect cells against heavy metal toxicity, particularly manganese [11, 12] [13]. Therefore, we examined whether HeLa cells stably overexpressing Atp13a2 would be more resistant to manganese toxicity. Accordingly, we treated control non-overexpressing or Atp13a2-overexpressing cell cultures with increasing concentrations of manganese chloride (MnCl_2_) for 16- or a 24-hour period and then quantified cell death in the cultures by Hoechst staining (Fig 3A and 3B). As expected, normal HeLa cells exposed to manganese displayed an increase in cell death compared to cells that were not treated with the heavy metal. By contrast, Atp13a2-overexpressing cells were significantly more resistant to manganese-induced cell death as evident by lower cell death at all time points and concentrations tested. These results suggest increased expression of Atp13a2 protects HeLa cells from manganese-induced toxicity.

### Overexpression of Atp13a2 increases autophagic flux and maturation of autophagosomes

**Fig 4:**
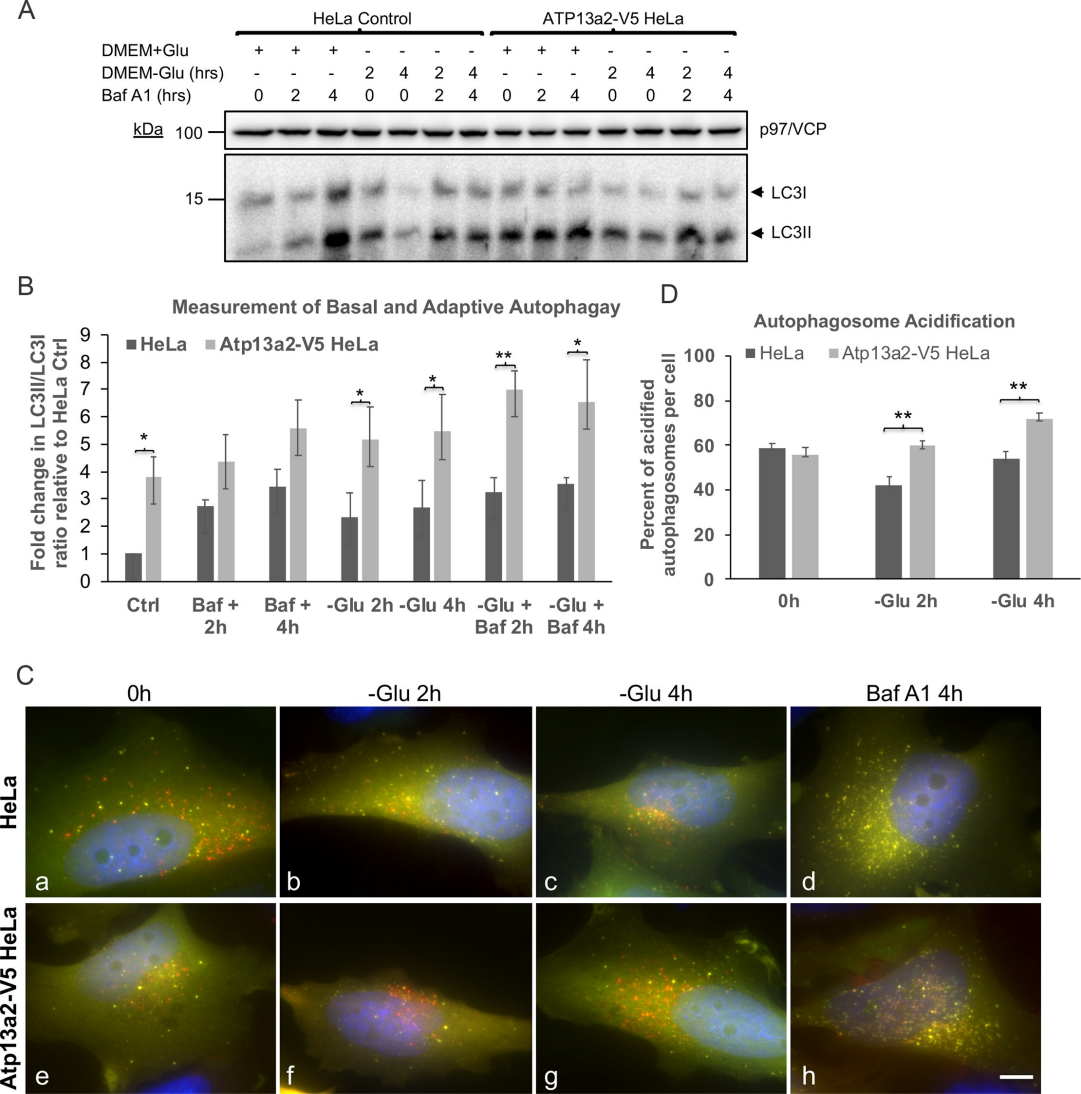
Increase in adaptive autophagy and autophagosome acidification in cells overexpressing Atp13a2. (A) Cells from the normal HeLa line or the line stably expressing Atp13a2-V5 were plated at equal density and the cultures were grown in normal glucose containing medium (+Glu) or shifted to media lacking glucose (-Glu) for the indicated times. Bafilomycin A1 was added to some of the cultures as indicated to block autophagy. Equal amounts of protein from the cultures were immunoblotted for LC3 and p97/VCP, which was used as a loading control. The position of the immature LC3I and lipidated mature LC3II protein are indicated by arrows. (B) Quantification of the ratio of LC3II to LC3I in three independent experiments (see S1 Table). (C) HeLa cells or HeLa cells stably expressing V5-tagged Atp13a2 were grown on coverslips in glucose-containing DMEM medium and transfected with the pDest-GFP-mCherry-LC3B reporter plasmid. The next day the cells were shifted to glucose-free DMEM medium and cultured with, or without Bafilomycin A1, for either 0, 2 or 4 h. The cells were then fixed and stained with DAPI. The individual GFP, mCherry and DAPI fluorescent images of the same cell is provided in S1 Fig, and the result of merging of the three individual images shown for the HeLa (a-d) and Atp13a2-V5 (e-h) cell lines. Bar, 10 μm. (D) Quantification of the percent of acidified autophagosomes per cell using the GFP-mCherry-LC3B reporter construct. (B, D) * indicate statistical differences determined by Two-way ANOVA analysis (*p < 0.05, **p < 0.01).

Because HeLa cells overexpressing Atp13a2-V5 were more resistant to starvation-induced cell death we examined if the cells had any alteration in either basal or adaptive autophagy. Basal or constitutive autophagy refers to the underlying autophagy that exists under normal growth conditions, whereas adaptive autophagy is the response elicited following limitation of nutrients, such as energy [32, 33]. We measured both forms of autophagy by adding Bafilomycin A1, an inhibitor that blocks autophagosome maturation, to HeLa cultures grown in normal glucose-containing DMEM medium for different lengths of time (to measure basal autophagy), or after switching the medium to one lacking glucose (to measure adaptive autophagy). Lysates from the cultures were probed for changes in accumulation of the ratio of LC3II to LC3I protein, to measure alterations in autophagy (Fig 4A). Measurement of the autophagic flux by these experiments indicated that basal autophagy in the Atp13a2-V5 cell line was not significantly different to the parental cell line (Fig 4B). By contrast, adaptive autophagy was found to be significantly elevated in the Atp13a2-V5 cell line compared to the parental HeLa line (Fig 4B and S1 Table).

To validate these findings, we examined whether autophagsome maturation was altered in the Atp13a2-V5 cell line by using the tandem-tagged GFP-mCherry-LC3B fluorescent reporter construct. As first reported by Pankiv et al. [30], autophagosomes in cells expressing the GFP-mCherry-LC3B reporter are distinguished by exhibiting both GFP and mCherry fluorescence (their combined color produces a yellow signal), whereas upon acidification into autolysosomes the GFP fluorescence is quenched resulting in more prominent mCherry fluorescence. We transfected cultures made from HeLa and Atp13a2-V5 cell lines with the GFP-mCherry-LC3B reporter construct and quantified autophagosome acidification (percent of mCherry puncta that lacked GFP fluorescence relative to the total number of mCherry puncta) in cells before and following 2 and 4 h after switching the culture medium to one lacking glucose (to induce adaptive autophagy) (Fig 4C). The quantification indicated that acidification of autophagosomes was higher in the Atp13a2-V5 cell line at both 2 and 4 h following glucose deprivation compared to the normal HeLa cell line (Fig 4C and 4D and S1 Fig). As expected, acidification of autophagosomes was blocked upon incubation of the cells with Bafilomycin A1, as shown by accumulation of mainly yellow puncta (Fig 4C, panels d and h).

### Overexpression of human Atp13a2 in *C*. *elegans* protects dopamine neurons against degeneration caused by manganese toxicity

**Fig 5:**
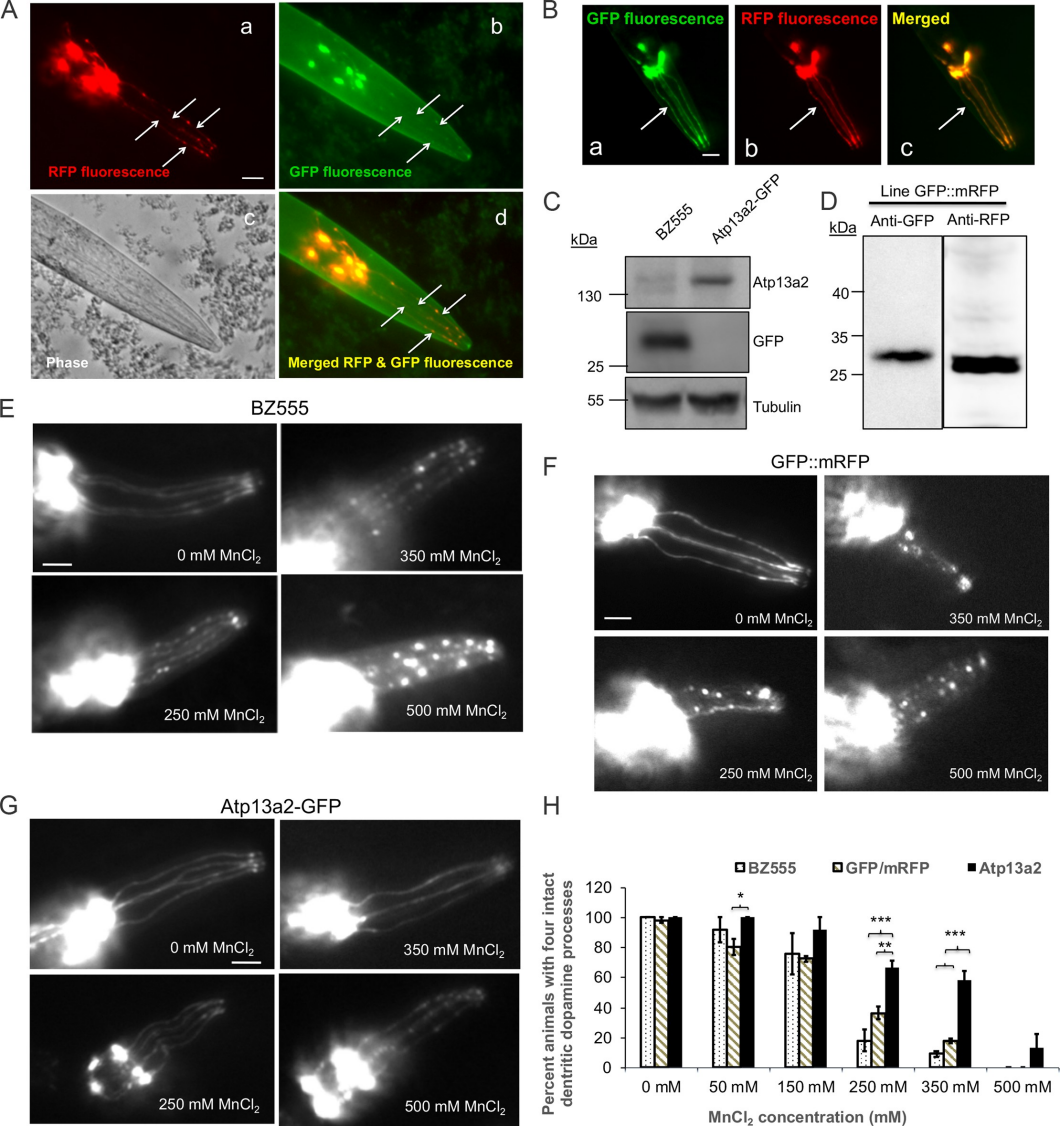
Nematodes expressing GFP-tagged Atp13a2 exhibit higher resistance to manganese induced dopamine neuron degeneration. (A) Double fluorescence microscopy demonstrating visual detection of RFP and GFP fluorescence in dopamine neuron processes (indicated with arrows) in the head of the *C*. *elegans* line coexpressing dat-1p:mRFP and dat-1p:Atp13a2-GFP. The phase contrast image of the head region is also shown, together with the image produced after merging the red and green fluorescent images. Because of its brighter fluorescence the RFP fluorescence was used to monitor dopamine neuron degeneration after manganese exposure. Bar, 25 μm. (B) Fluorescent images of the head region of the nematode line coexpressing dat-1 promoter driven expression of GFP (a) and mRFP (b) and the result of merging the two fluorescent proteins (c). The arrow shows clear colocalization of GFP and mRFP fluorescence in dopamine neurons. (C) Immunoblots of *C*. *elegans* lysates prepared from the BZ555 and Atp13a2-GFP lines with antibodies specific for Atp13a2, GFP or tubulin. The immunoblots confirmed expression of the appropriate size Atp13a2-GFP protein and GFP proteins in the two different lines. (D) Immunoblot of the GFP::mRFP coexpressing line for GFP and RFP expression. (E, F and G) Representative images showing differential sensitivity of dopamine neurons in the nematode BZ555 line (E) GFP and mRFP expressing nematodes (F) and the Atp13a2-GFP-expressing nematode line (G) to acute exposure for 1 h with different concentrations of MnCl_2_. Bar, 25 μm. (H) Quantification of dopamine neuron degeneration in the BZ555, GFP::mRFP and Atp13a2-GFP expressing nematode lines. The quantification shown is the mean ±SDM of three independent experiments. At least 20 nematodes were counted for each exposure condition. Nematodes expressing Atp13a2-GFP exhibit significantly higher resistance to manganese-induced neurodegeneration at 250 and 350 mM MnCl_2_ treatment (*p < 0.05, **p < 0.01, ***p < 0.001).

Because PD is associated with loss of dopamine neurons, we next determined whether overexpression of Atp13a2 would protect dopamine neurons from neurodegeneration. To examine this possibility, we turned to *C*. *elegans*, as it provides a simple yet powerful model system to study toxicity and/or neurodegeneration of dopamine neurons [34]. A previous study had suggested that overexpression of the *C*. *elegans* W08D2.5 protein, the putative homolog of Atp13a2 protein that is only 34% identical to the human protein, protects dopamine neurons against alpha-synuclein toxicity [12]. To test whether Atp13a2 has a protective function we generated a transgenic *C*. *elegans* line expressing green fluorescent protein (GFP)-tagged human Atp13a2^Isoform-1^ protein and soluble monomeric red fluorescent protein (mRFP) in dopamine neurons, both driven by the dat-1 promoter [34]. An additional line expressing untagged GFP and mRFP was also generated. Fluorescent microscopy of live nematodes revealed weak, but visible expression of the GFP-tagged Atp13a2 protein exclusively in dopamine neurons, which colocalized with the brighter mRFP fluorescence in the same neurons (Fig 5A, panels a-d). The line expressing untagged GFP and mRFP had bright fluorescence of both proteins in dopamine neurons (Fig 5B). Immunoblotting of lysates with an anti-Atp13a2 specific antibody confirmed expression of the appropriate size human Atp13a2 protein in the Atp13a2-GFP transgenic line, but not in nematodes expressing GFP alone (Fig 5C). Additional immunoblots also confirmed expression of the appropriate size GFP and mRFP proteins in the line containing both expressed proteins (Fig 5D).

We next examined whether overexpression of human Atp13a2 in dopamine neurons increased resistance to manganese toxicity. *C*. *elegans* lines BZ555 and GFP::mRFP that express either GFP only, or GFP and mRFP, respectively, in dopamine neurons, were used as controls. For the Atp13a2-expressing nematodes, coexpressed soluble mRFP was used as a marker to monitor neurodegeneration. The manganese toxicity studies for all three lines were conducted by exposing synchronized L1-stage nematodes for each line to acute 1-hour exposure with increasing concentrations of MnCl_2_ and scored for the extent of dopamine neuron degeneration (Fig 5E, 5F and 5G). Neurodegeneration was scored in the nematodes by determining whether any one of the four CEP dopamine neuron dendritic processes in the head of the nematode were lost, or if they displayed signs of blebbing by fluorescent microscopy, as previously established [34].

Quantification of neurodegeneration revealed a concentration-dependent sensitivity of BZ555 nematodes to MnCl_2_ concentrations of 250 mM and above (Fig 5E and 5H and S2 Table). This sensitivity is lower than previously reported [35], but could be attributed to variation in scoring criteria and/or strain or culture conditions. Similar sensitivity to manganese toxicity was observed for the line expressing both GFP and mRFP (Fig 5F and 5H). By contrast, the nematode line overexpressing Atp13a2 were significantly more resistant to dopamine neuron degeneration at 250 and 350 mM MnCl_2_ (p<0.001) compared to the BZ555 line (Fig 5G and 5H). At 250 and 350 mM concentrations of MnCl_2,_ approximately 67% and 58% of nematodes overexpressing Atp13a2 were unaffected compared to only 18% and 9% in BZ555, and 36% and 17% in GFP::mRFP nematodes at the same two concentrations. This result demonstrates the importance of human Atp13a2^Isoform-1^ protein in conferring resistance to manganese toxicity in a living organism.

## Discussion

Here, we report on the cytoprotective properties conferred by the human Atp13a2^Isoform-1^ protein in HeLa cells and dopamine neurons of *C*. *elegans*. In both cell types we found overexpression of the protein confers resistance to manganese toxicity, suggesting the Atp13a2 protein plays an important role in cytoprotection against this heavy metal. Protein localization data suggests that this function is most likely conferred by the action of the protein in lysosomes [6]. In accord with its action in lysosomes, we found that cells overexpressing Atp13a2 were more resistant to starvation-induced cell death, a condition when lysosomal function becomes important for survival. Indeed, measurement of autophagosome acidification, where lysosomal function is vital, was increased in the HeLa cell line overexpressing Atp13a2 under conditions of adaptive autophagy, compared to the parental HeLa cell line. Taken together our results suggest that Atp13a2^Isoform-1^ protein plays an important role in protecting cells against adverse insults, and in particular to resistance to manganese toxicity.

The protective function of Atp13a2 against manganese toxicity has important implications for PD. Manganese plays an important role in the cell as a cofactor for various enzymes. However, high levels of manganese cause oxidative stress, mitochondria dysfunction, and apoptosis [36]. Our results show that overexpression of Atp13a2 confers higher tolerance of cells and organisms to manganese toxicity. We speculate that increased expression of Atp13a2 may enhance transport of manganese into the lysosome, thus protecting the cells from the toxic effects of the heavy metal in the cytosol. The idea that Atp13a2 may function as a manganese pump has been suggested before [10–13, 20]. As mentioned previously, manganese toxicity has been linked to the development of manganism [20], a PD-like syndrome, suggesting that maintaining manganese homeostasis is essential for cell survival. To our knowledge, only one other manganese P-type ATPase, the Golgi-localized ATP2C1 (SPCA1), has been identified in mammals. Human ATP2C1, as well as the *C*. *elegans* and yeast homolog PMR1, has shown to play an important role in protecting cells from manganese toxicity [37–41]. Thus, it is possible that Atp13a2-mediated transport of manganese into the lysosome contributes to maintaining manganese homeostasis in the cell and loss of function mutations in *ATP13A2* may result in manganese toxicity and disease.

A second, but not necessarily mutually exclusive possibility is that Atp13a2 overexpression enhances the robustness of lysosomes leading to proper maintenance of proteostasis. Support for this idea was strengthened by our findings that HeLa cells overexpressing Atp13a2 protein were significantly more resistant to cell death induced by nutrient deprivation. Lysosomes play a critical role during nutrient deprivation, when autophagy is activated. Specifically, they fuse with autophagosomes providing the hydrolytic enzymes critical for breakdown and recycling of cellular components. Consistent with this idea, we found adaptive autophagic flux and acidification of autophagosomes were both increased in the HeLa cells overexpressing Atp13a2 compared to normal HeLa cells. The exact reason by which Atp13a2 overexpression increases autophagosome acidification is unclear, but we speculate it may be either related to affecting the activity, fusion capacity, or the overall robustness of lysosomes. Thus, we speculate that overexpression of Atp13a2 makes lysosomes more potent or robust leading to the greater protective effect observed in our assays. Interestingly, studies have shown that loss of Atp13a2 in cells leads to a disruption in autophagic flux and or dysfunction of the endolysosomal pathway [9, 18, 23, 42]. The robustness of lysosomes exerted by ATP13a2 may extend to effects in proper handling of aggregate prone proteins in cells, such as α-synuclein. This idea is consistent with the findings that overexpression of Atp13a2 is protective against α-synuclein toxicity and loss of Atp13a2 exacerbates α-synuclein aggregation [10, 12, 15, 17, 18]. Additionally, *ATP13A2* KO mice were not only found to contain higher levels of manganese and iron accumulation in the brain following intraperitoneal injection of manganese chloride, compared to mice with normal ATP13a2 expression, but also increased α-synuclein aggregation and higher lipofusin material in the substantia nigra [10]. Thus, ATP13A2 may function both in protection to heavy metal toxicity as well as in protection of aggregation of proteins by proper maintenance of proteostasis. Finally, since dysfunctions in endolysosomal system have been linked to neurodegenerative disorders [27], future studies of Atp13a2 may yield valuable information on its function in health and disease.

## Supporting information

S1 Table Table of two-way ANOVA data for Fig 4B. Two-way ANOVA analysis of the LC3II to LC3I changes after normalization for p97 loading shown in Fig 4A and 4B.
(TIF)

S2 Table Table of two-way ANOVA data for Fig 5H. Two-way ANOVA analysis of the dendritic dopamine processes in animals following treatment with the different concentrations of MnCl_2_ the results of which are shown in Fig 5H.
(TIF)

S1 Fig Cells overexpressing Atp13a2 show increased autophagosome acidification during adaptive autophagy. (A-C). Images of the individual GFP, mCherry, DAPI and their resulting combined image used to construct the panels shown in Fig 4C. Bar, 10 μm.
(TIF)
