# Supplementary figures and images for: Overexpression of human Atp13a2Isoform-1 protein protects cells against manganese and starvation-induced toxicity
Source: PLoS One. 2019 Aug 8;14(8):e0220849. doi: 10.1371/journal.pone.0220849 (PMC6687281; doi:10.1371/journal.pone.0220849)

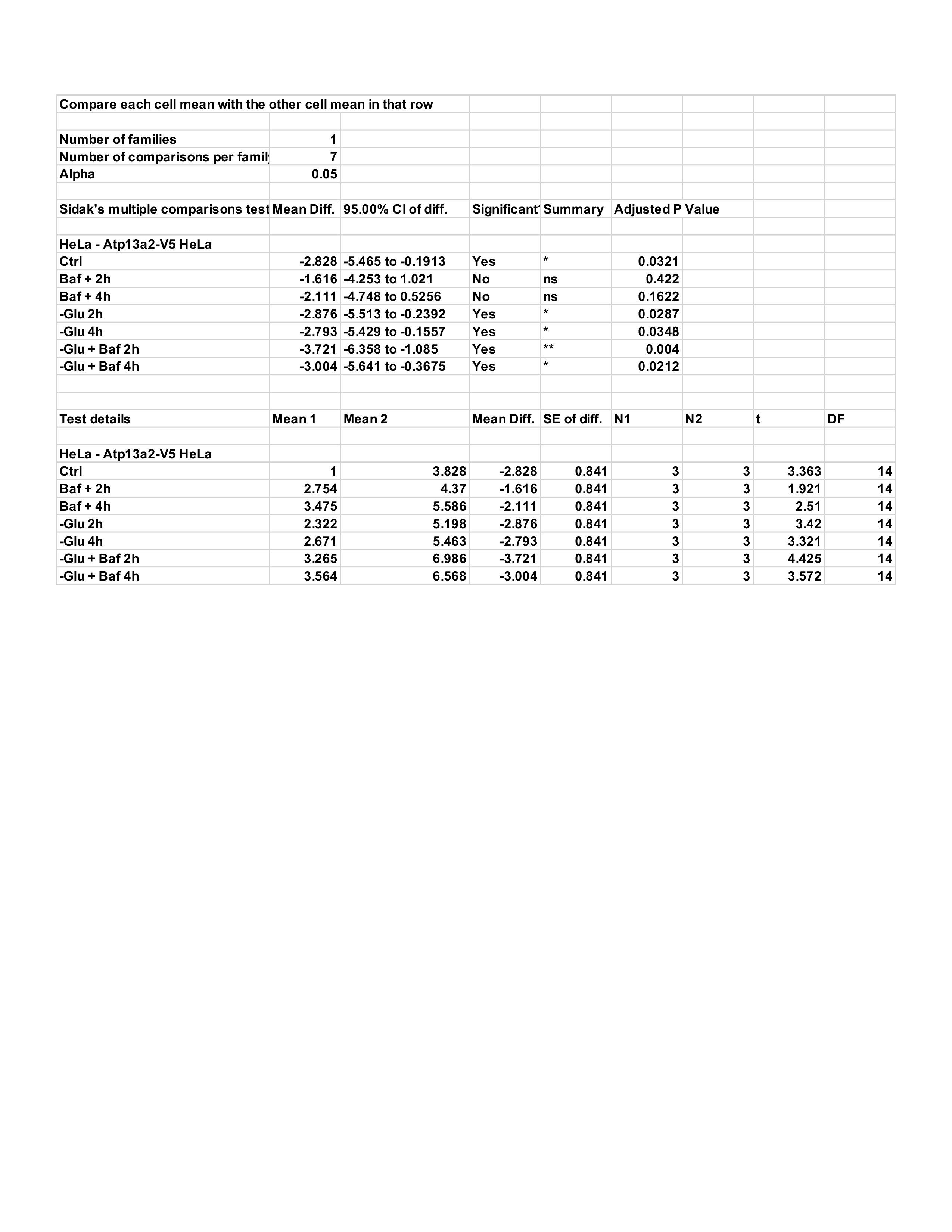

Supplement: S1 Table — Two-way ANOVA analysis of the LC3II to LC3I changes after normalization for p97 loading shown in Fig 4A and 4B. (TIF) [file pone.0220849.s001.tif]

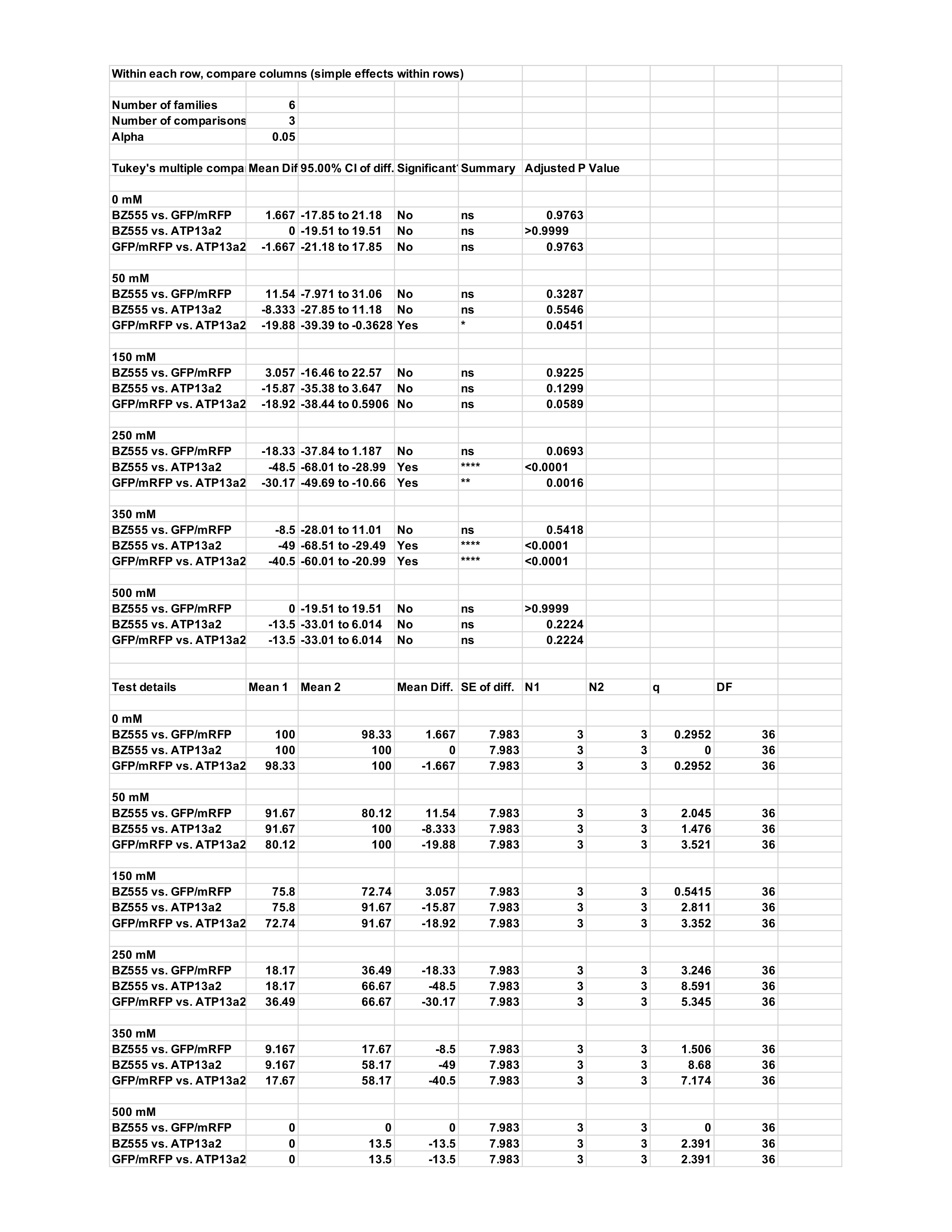

Supplement: S2 Table — Two-way ANOVA analysis of the dendritic dopamine processes in animals following treatment with the different concentrations of MnCl2 the results of which are shown in Fig 5H. (TIF) [file pone.0220849.s002.tif]

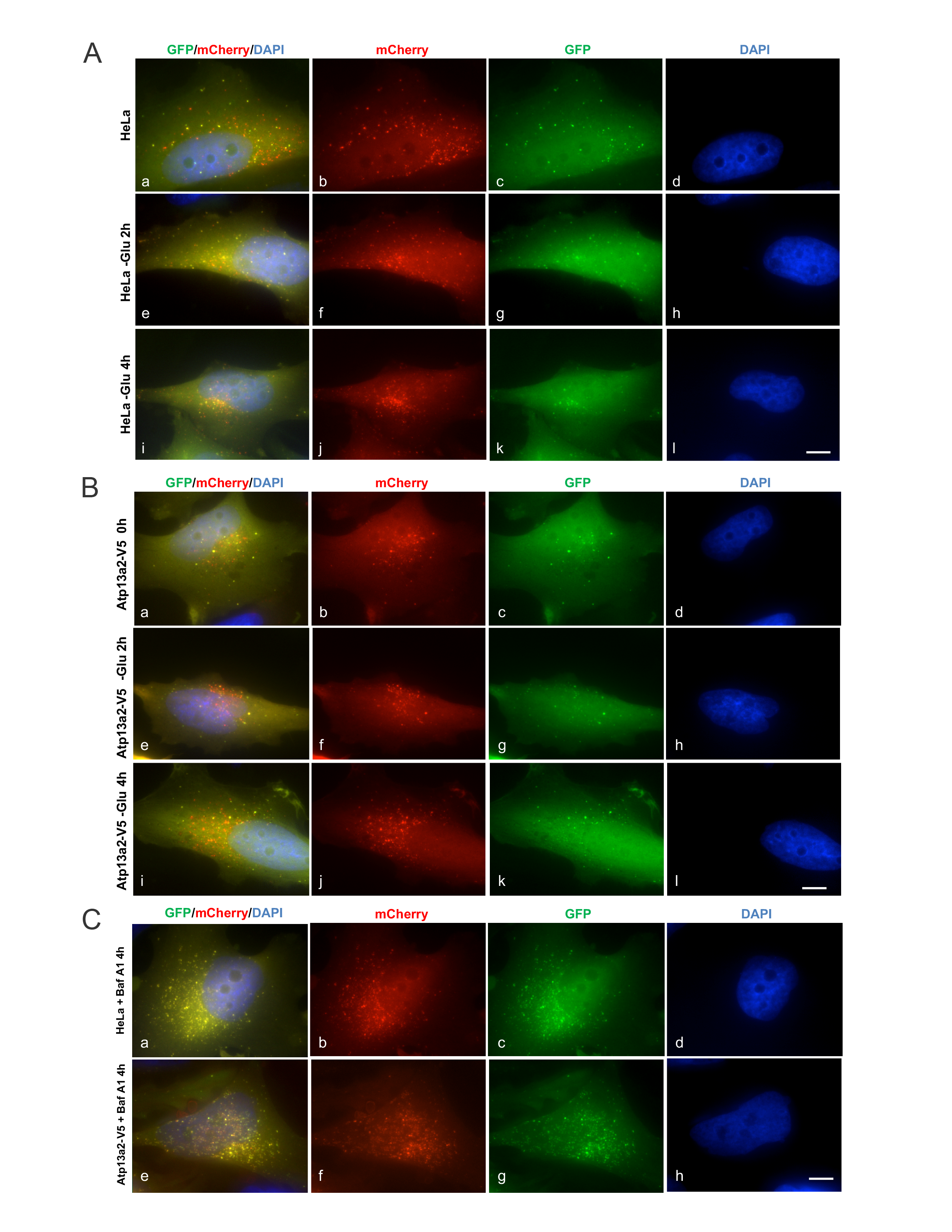

Supplement: S1 Fig — (A-C). Images of the individual GFP, mCherry, DAPI and their resulting combined image used to construct the panels shown in Fig 4C. Bar, 10 μm. (TIF) [file pone.0220849.s003.tif]
